# Supplementary material for: The Essential Oil Composition of Trachymene incisa Rudge subsp. incisa Rudge from Australia
Source: Plants (Basel). 2021 Mar 23;10(3):601. doi: 10.3390/plants10030601 (PMC8005043; doi:10.3390/plants10030601)
Supplement: Supplementary file 1 [file plants-10-00601-s001.zip › Supplementary-Table S1-Peaks-I.pdf]

Table 1. Essential oil composition (%) of *Trachymene incisa* subsp. *incisa* variants.

Peak Nº#	KIa.	KI[1]	KIb	KI[2]	Compound	C. type	T.in.I$_1$	T.in.I$_2$	T.in.I$_3$	T.in.II$_4$	T.in.II$_5$	I.M.
1	1007	1025	932	936	α-pinene	MH	7.2		**19.2**	t	0.1	KI, MS
2	1034	1061	945	949	α-fenchene	MH			0.8			KI, MS
3	1041	1068	946	950	camphene	MH			t			KI, MS
4	1084	1110	974	978	β-pinene	MH	0.4		2.2	t	0.1	KI, MS
5	1105	1122	969	973	sabinene	MH						KI, MS
6	1127	1146	1008	1011	δ-3-carene	MH			1.8			KI, MS
7	1139	1167	1002	1004	α-phellandrene	MH			1.2			KI, MS
8	1141	1160	988	989	myrcene	MH	t					KI, MS
9	1157	1177	1014	1017	α-terpinene	MH						KI, MS
10	1176	1198	1024	1029	limonene	MH	t		0.8			KI, MS
11	1178	1209	1025	1030	β-phellandrene	MH			t			KI, MS
12	1181	1211	1026	1031	1,8-cineole	OM	t		9.0			KI, MS
13	1218	1234	1032	1038	*Z*-β-ocimene	MH			t			KI, MS
14	1224	1245	1054	1059	γ-terpinene	MH			t			KI, MS
15	1226	1250	1044	1048	*E*-β-ocimene	MH			t			KI, MS
16	1249	1270	1020	1024	*p*-cymene	MH	t	t	0.6	t	t	KI, MS
17	1260	1282	1086	1086	terpinolene	MH		t	0.4	0.9		KI, MS
18	1320	1237	989	986	6-methylhept-5-en-2-one	O		t	0.2	t	t	KI, MS
19	1459	1469	1335	1337	δ-elemene	SH	0.3	0.3		t		KI, MS
20	1462	1491	1374	1376	α-copaene	SH	t	3.4		t		KI, MS
21	1510	1541	1387	1387	β-cubebene	SH		0.6		t		KI, MS
22	1523	1559	1411	1414	α-*cis*-bergamotene	SH			0.6	0.4		KI, MS
23	1543	1575	1432	1435	α-*trans*-bergamotene	SH		0.2	2.1	t		KI, MS
24	1560	1590	1389	1390	β-elemene	SH	0.8	2.9	0.4	t		KI, MS
25	1563	1598	1417	1420	β-caryophyllene	SH	2.5	5.4	1.2	**10.8**	**10.4**	KI, MS
26	1570	1620	1439	1441	aromadendrene	SH	1.5	0.6	0.5	5.6	5.0	KI, MS
27	1572	1629	1509	1504	α-bulnesene	SH	0.5		t	1.2	0.9	KI, MS
28	1632	1649	1458	1460	*allo*-aromadendrene	SH	0.2	0.4	0.4	0.4	0.4	KI, MS
29	1644	1664	1454	1456	(*E*)-β-farnesene	SH		0.3	1.4	0.6		KI, MS
30	1653	1666	1452	1453	α-humulene	SH	0.5	1.5	0.3	1.8	1.8	KI, MS
31	1658				$C_{15}H_{24}$	SH	0.4	0.3	0.7			KI, MS
32	1661	1696	1496	1492	viridiflorene	SH	1.9	0.8	0.6	5.4	4.7	KI, MS
33	1670	1708	1484	1480	germacrene-D	SH	1.4	2.4	1.5	0.5		KI, MS
34	1680	1705	1505	1512	γ-bisabolene	SH			**27.4**			KI, MS
35	1697	1717	1489	1486	β-selinene	SH	**36.7**	**11.9**	0.5	1.8		KI, MS
36	1706	1734	1500	1494	bicyclogermacrene	SH	**28.0**	**21.5**	7.5	**34.7**	**24.4**	KI, MS

37	1722	1763	1513	1513	γ-cadinene	SH	t					KI, MS
38	1724	1755	1522	1521	δ-cadinene	SH	t	9.0	0.5	1.5	1.7	KI, MS
39	1727	1788	1495	1531	cis-cadina-1,4-diene	SH		0.3		t		KI, MS
40	1734	1744	1505	1504	(E,E)-α-farnesene	SH	t	1.4	2.6		1.6	KI, MS
41	1749	1773	1479	1482	ar-curcumene	SH		t	t	0.7	1.1	KI, MS
42	1886	1986	1582	1580	caryophyllene oxide	OS	1.6	2.3	0.2	1.0	**12.8**	KI, MS
43	1958	2039	1602	1582	ledol	OS	0.2	0.2	0.1	0.5	t	KI, MS
44	2016	2067	1645	1636	cubenol	OS	t	0.9	0.2	0.8	1.5	KI, MS
45	2018	2074	1595	1588	cubeban-11-ol	OS	0.7	0.4	0.4	1.6	1.1	KI, MS
46	2034	2082	1590	1582	globulol	OS	2.0	1.5	1.3	7.0	6.9	KI, MS
47	2044	2090	1592	1591	viridiflorol	OS	1.5	1.1	0.8	3.9	3.5	KI, MS
48	2066				$C_{15}H_{26}O$	OS	0.5	0.3	0.3	2.2	2.0	KI, MS
49	2074				$C_{15}H_{26}O$	OS	0.4	0.6	0.3	1.3	1.0	KI, MS
50	2078				$C_{15}H_{26}O$	OS	0.2	0.3				KI, MS
51	2086	2127	1577	1576	spathulenol	OS	4.2	11.2	2.9	6.7	7.5	KI, MS
						Total	**93.5**	**81.7**	**91.0**	**91.1**	**88.4**	
Monoterpene Hydrocarbon (MH)						16	7.6	0	27.0	0.9	0	
Oxigenated Monoterpene (OM)						1	0	0	9.0	0	0	
Sesquiterpene Hydrocarbon (SH)						23	74.8	63.0	48.3	65.4	52.0	
Oxigenated Sesquiterpene (OS)						10	11.1	18.7	6.5	24.8	36.3	
Other (O)						1	0	0	0.2	0	0	
Total						**51.0**	**93.5**	**81.7**	**91.0**	**91.1**	**88.2**	

C. type = Compound type; MH = monoterpene hydrocarbon, OM = oxygenated monoterpene, SH = sesquiterpene hydrocarbon, SO = oxygenated sesquiterpene, O = Other; t = traces (<0.1%); KIa and KIb = linear retention index relative to n-alkanes on DB-Wax column or DB-5 column respectively; KI^1 and KI^2= literature linear retention index on DB-Wax column [48] and DB-5 column respectively [47]; T.In. = *Trachymene incisa* subsp. *incisa*; I and II = typical glabrous and hairy variant respectively; I.M. = Identification method.
