# Supplementary figures and images for: The Essential Oil Composition of Trachymene incisa Rudge subsp. incisa Rudge from Australia
Source: Plants (Basel). 2021 Mar 23;10(3):601. doi: 10.3390/plants10030601 (PMC8005043; doi:10.3390/plants10030601)

**T.in.I<sub>1</sub>**

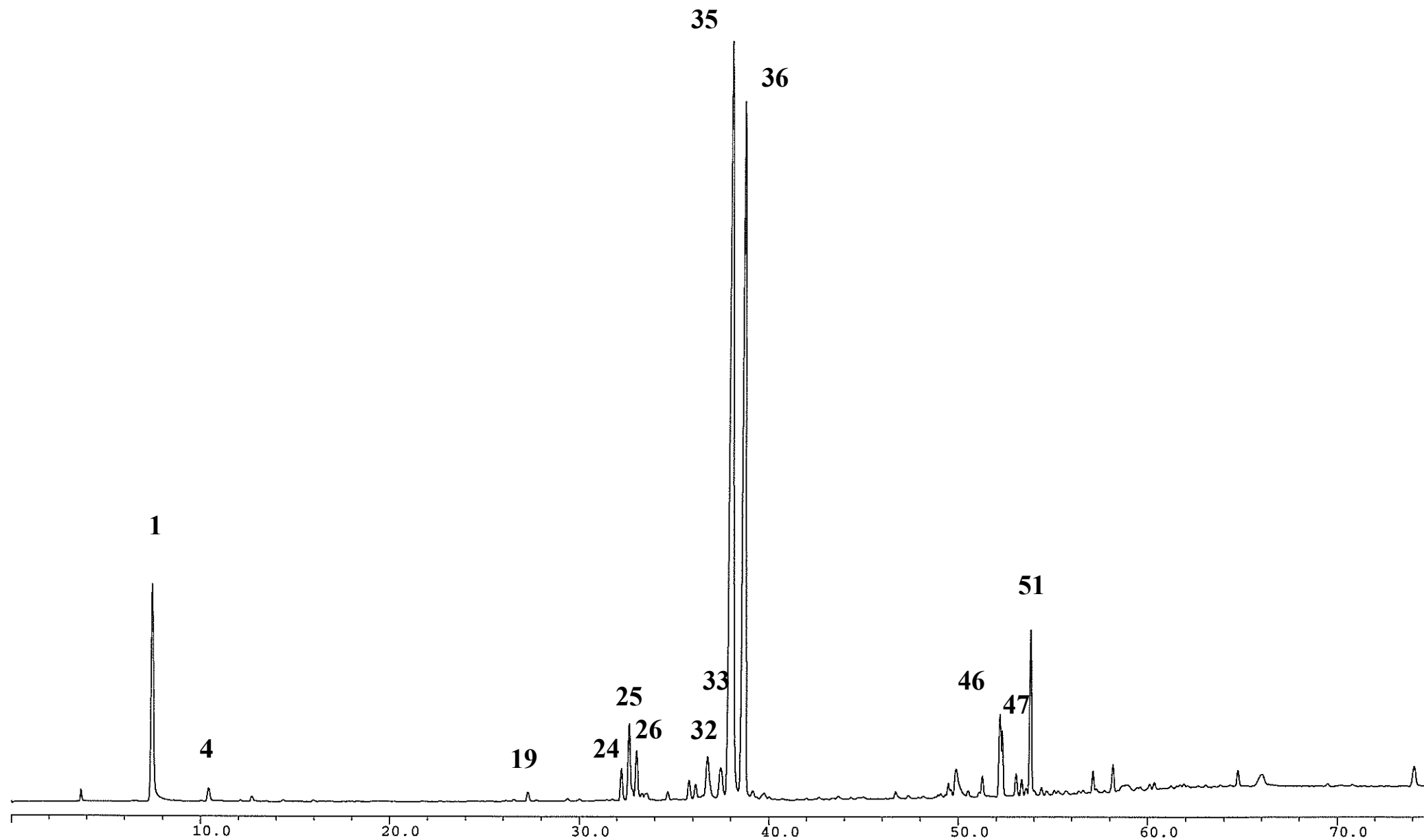

**T.in.I<sub>2</sub>**

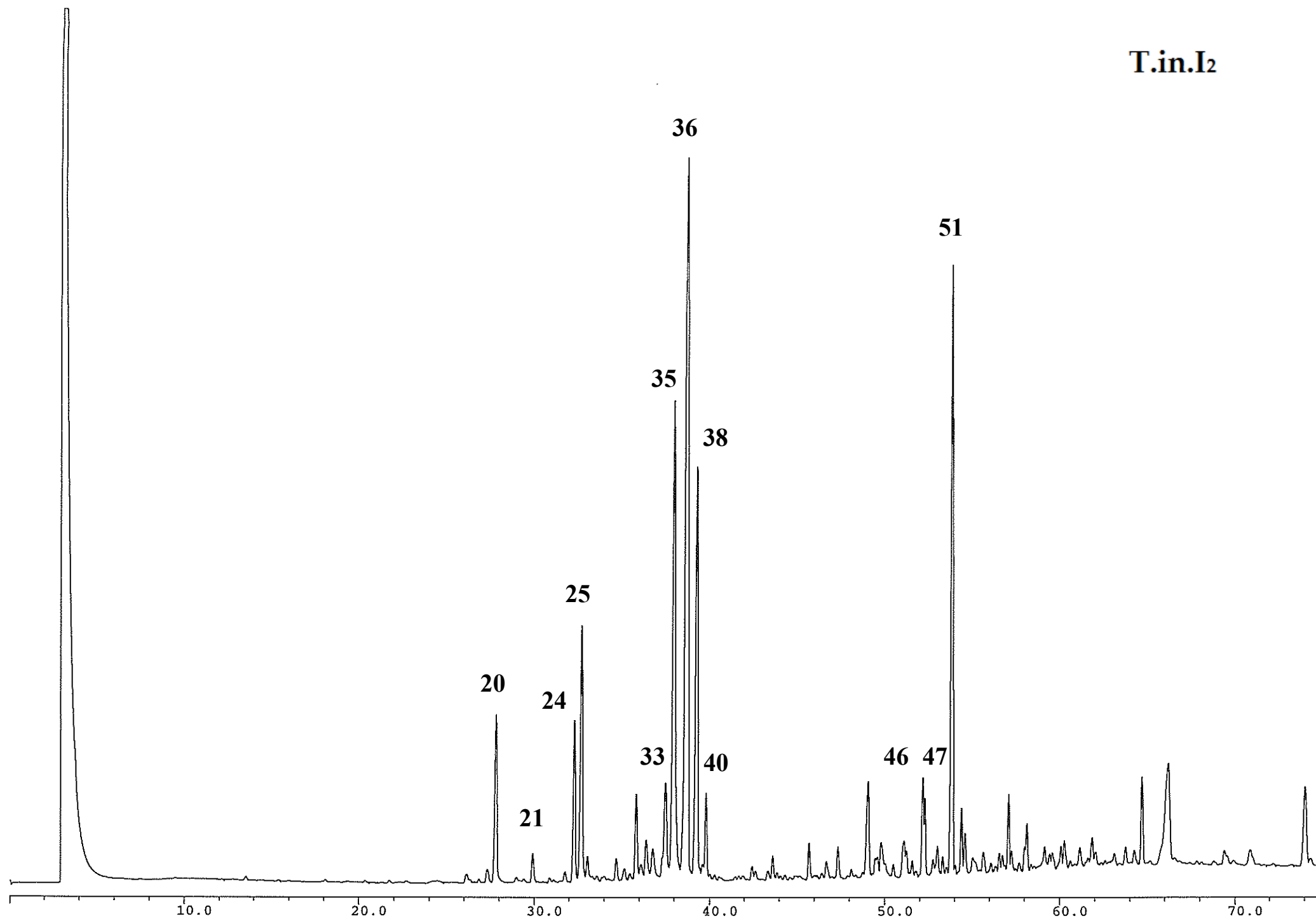

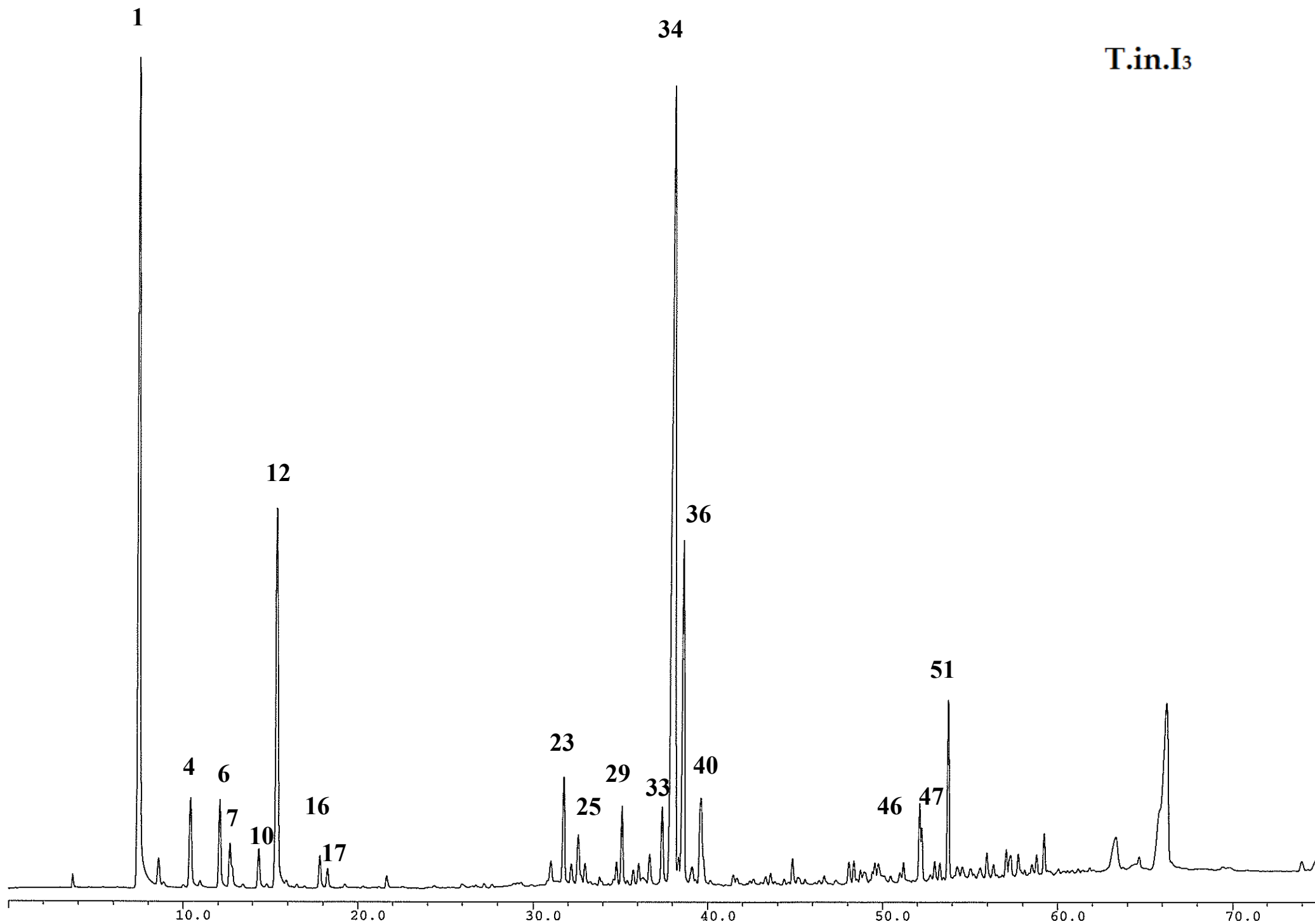

**T.in.II<sub>4</sub>**

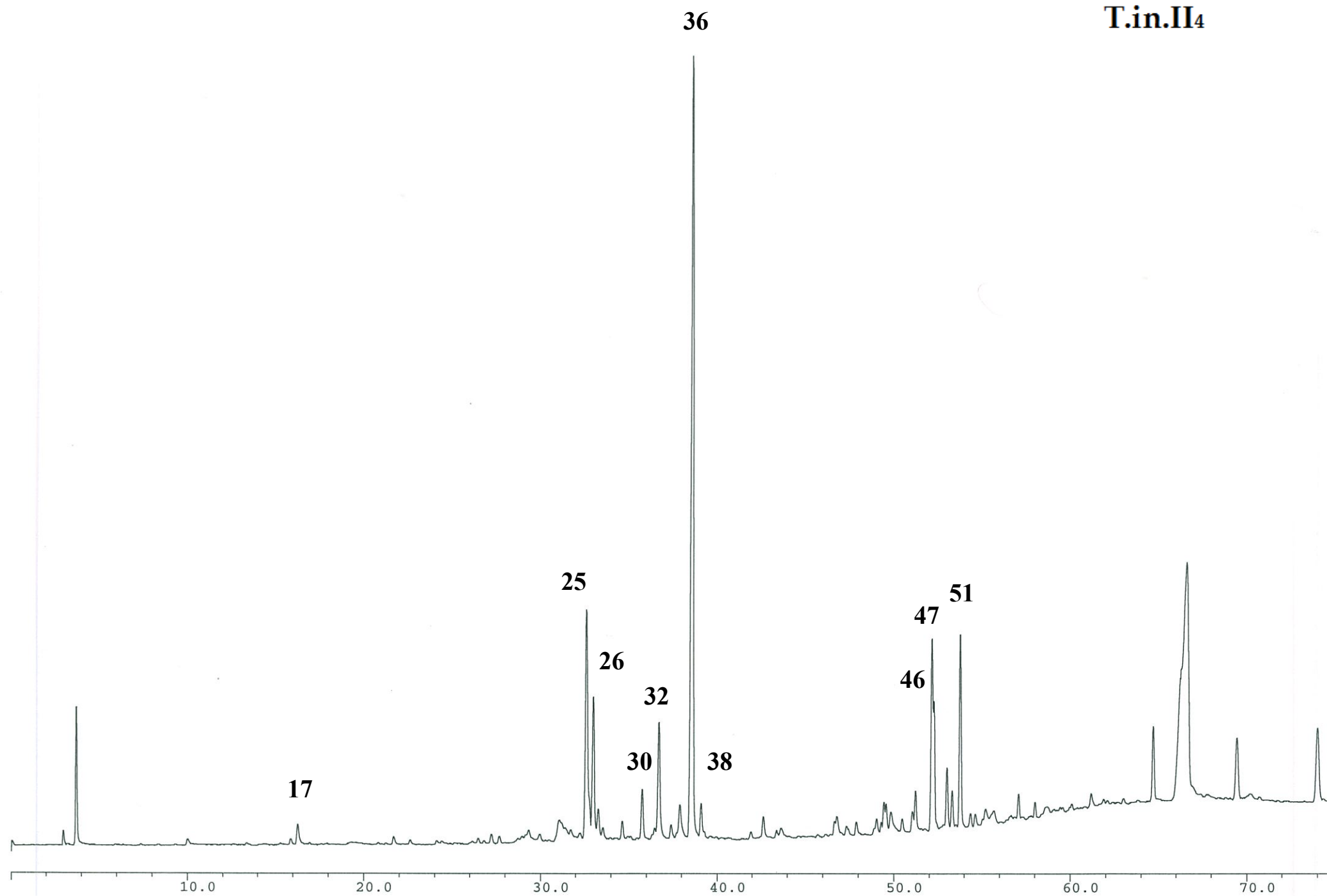

**T.in.II<sub>5</sub>**

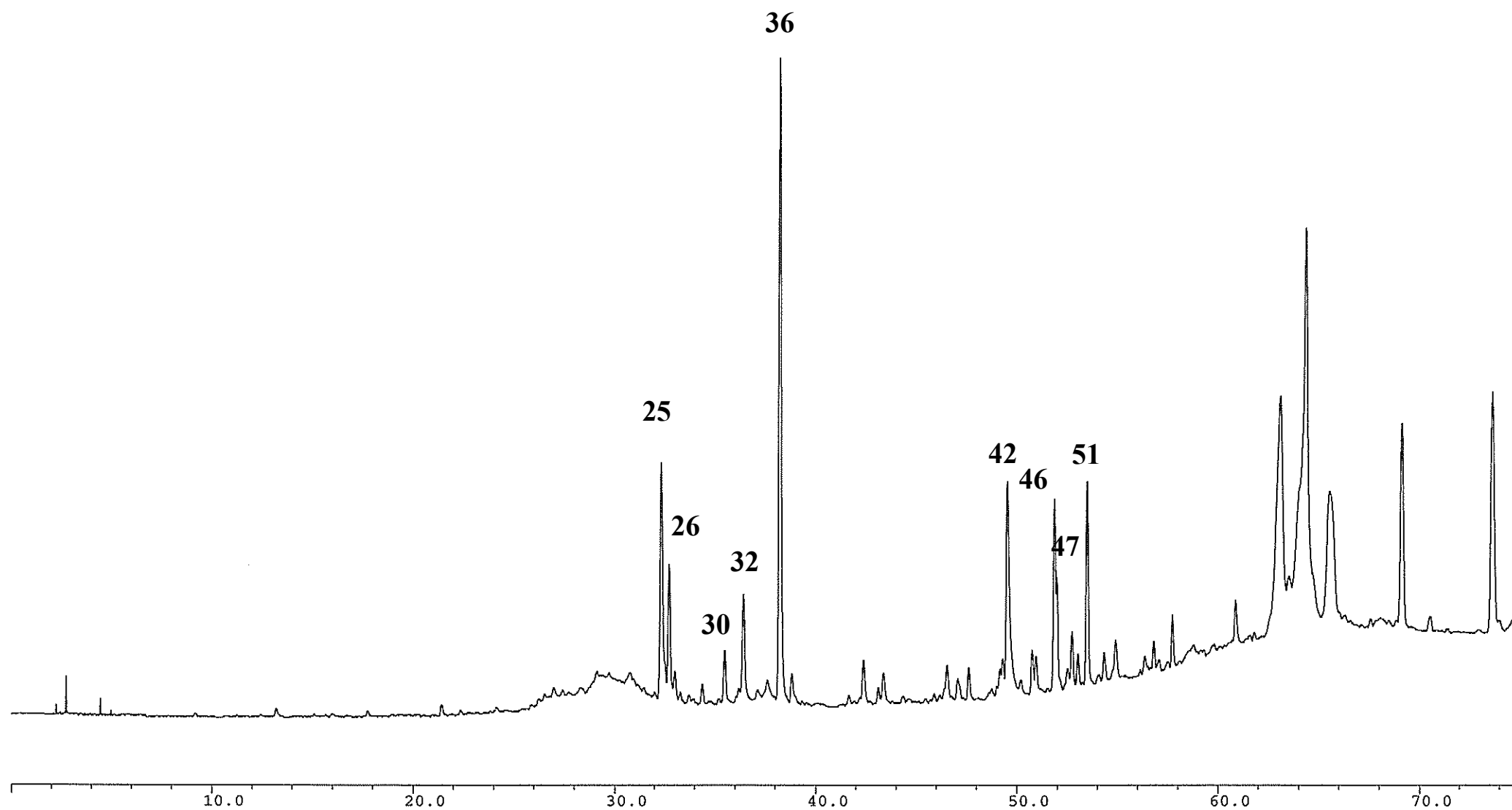

Supplement: Supplementary file 1 [file plants-10-00601-s001.zip › Supplementary-Figure S1.pdf]
